# The Rules of Engagement: Do Microglia Seal the Fate in the Inverse Relation of Glioma and Alzheimer’s Disease?

**DOI:** 10.3389/fncel.2019.00522

**Published:** 2019-11-20

**Authors:** Mathilde Cheray, Vassilis Stratoulias, Bertrand Joseph, Kathleen Grabert

**Affiliations:** ^1^Toxicology Unit, Institute of Environmental Medicine, Karolinska Institutet, Stockholm, Sweden; ^2^Neuroscience Center, Helsinki Institute of Life Science, University of Helsinki, Helsinki, Finland

**Keywords:** disease-associated microglia, glioma, Alzheimer’s disease, inverse correlation, risk genes

## Abstract

Microglia, the immune cells of the brain, play a major role in the maintenance of brain homeostasis and constantly screen the brain environment to detect any infection or damage. Once activated by a stimulus, microglial cells initiate an immune response followed by the resolution of brain inflammation. A failure or deviation in the housekeeping function of these guardian cells can lead to multiple diseases, including brain cancer and neurodegenerative diseases such as Alzheimer’s disease (AD). A small number of studies have investigated the causal relation of both diseases, thereby revealing an inverse relationship where cancer patients have a reduced risk to develop AD and *vice versa*. In this review, we aim to shed light on the role of microglia in the fate to develop specifically glioma as one type of cancer or AD. We will examine the common and/or opposing genetic predisposition as well as associated pathways of these diseases to unravel a possible involvement of microglia in the occurrence of either disease. Lastly, a set of guidelines will be proposed for future research and diagnostics to clarify and improve the knowledge on the role of microglia in the decision toward one pathology or another.

## Introduction

The central nervous system (CNS) can be affected at any stage in life by numerous neurological disorders and neurodegenerative diseases. Microglia, as key innate immune cells of the CNS, are the first responders and able to recognize an abundance of factors that could compromise the CNS. For the protection of the brain, microglia can trigger a vigorous immune and inflammatory response and therefore puts them at the center of neurological conditions (Biber et al., 2007; Wake et al., 2009; Chen and Trapp, 2016). The responsiveness of microglia in various diseases has been studied in great detail. Collectively, these studies revealed the high plasticity of microglia and associated regulative processes evident by the acquisition of regionally distinct spatio-temporal phenotypes throughout life as adaptation to their local environment (Shemer et al., 2015; Grabert et al., 2016; Ayata et al., 2018; Cheray and Joseph, 2018; Hammond et al., 2019; Masuda et al., 2019; Stratoulias et al., 2019). Overall, microglia phenotype is influenced by a variety of factors including ontogeny (Ginhoux et al., 2010; Kierdorf et al., 2013; Matcovitch-Natan et al., 2016), sex (Guneykaya et al., 2018; Villa et al., 2018; Hammond et al., 2019), location (de Haas et al., 2008; Grabert et al., 2016; Masuda et al., 2019), disease (Bisht et al., 2016; Keren-Shaul et al., 2017), and age (Grabert et al., 2016; Galatro et al., 2017). Aging is a natural, yet highly complex process, in which every part of an organism gradually declines. It is a devious and multi-layered progression affecting both the gene and protein level (e.g., DNA replication errors, epigenetic changes, protein misfolding) as well as cellular biochemistry and bioenergetics, which overall leads to an impairment of tissue homeostasis and function (López-Otín et al., 2013). Glioma and AD share aging as a common risk factor. In light of microglial plasticity, it is highly interesting that a number of studies suggest an inverse correlation between AD and cancer in general implying microglia may be set or contribute to an environment, which does not allow to change direction. Considering the vast advances made in microglia biology in glioma and AD, very little is known about the involvement of these immune sentinels to regulate or contribute to this intriguing relation of developing one yet being protected from acquiring the other pathology.

In this short review, we will depict the findings of the role and functionality of microglia in primary brain tumors and AD. Furthermore, we will provide a brief overview of longitudinal studies describing the inverse relation of both diseases. We then endeavor to discuss and elucidate in microglia reported risk genes and associate pathways to establish a link and/or cause in the fate toward either pathology.

## Microglia in Glioma

**FIGURE 1:**
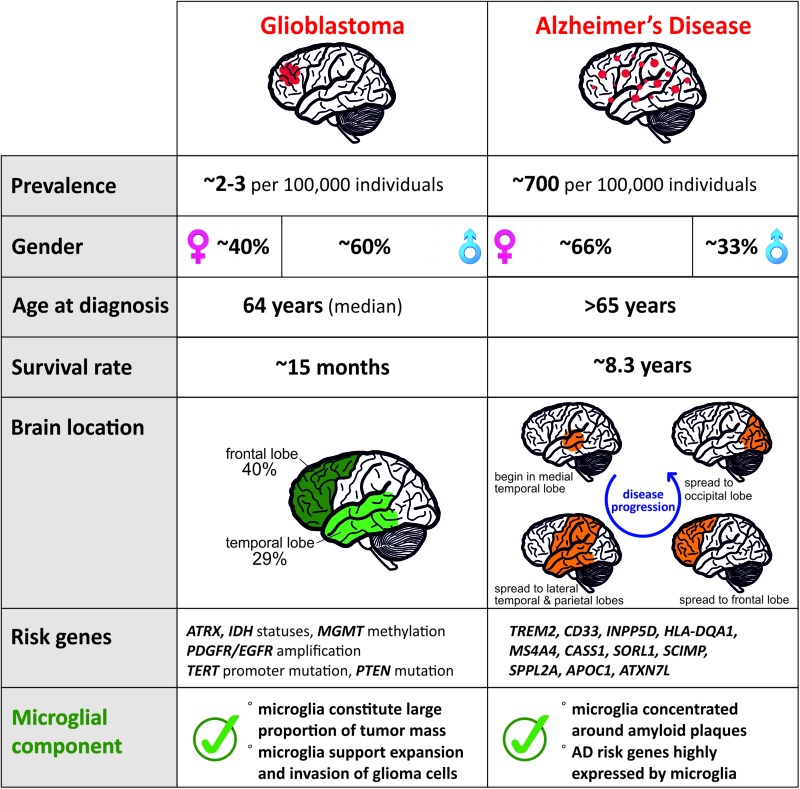
Comparison of distinct features for Glioblastoma and AD reveals contrasting pathologies and involvement of microglia.

Glioma, the most frequent of primary CNS tumors in adults, are divided into subtypes by the World Health Organization (WHO). While the low grade astrocytoma (grade I or II) are treatable when diagnosed early, high-grade glioma like Glioblastoma (grade IV) have a median survival limited to ∼15 months (Stupp et al., 2005). To date, it remains unclear how or why this type of cancer is initiated. The classification of different grades of glioma has allowed a better understanding on the different genetic actors involved in the progression of the disease. The mutation status of the isocitrate dehydrogenase (*IDH*) gene and the amplification of platelet-derived growth factor receptor (*PDGFR*) and epidermal growth factor receptor (*EGFR*) is of importance in the diagnosis and prognosis of glioma (Verhaak et al., 2010; Leu et al., 2013; The Cancer Genome Atlas Research Network, 2015). Additionally, the status of the deletion 1p/19q will differentiate grade I from grade II tumors (Leeper et al., 2015). New tumor microenvironment-derived subtypes of GBM (Wang et al., 2017) and new markers related to epigenetic changes, such as the promoter methylation status of the DNA repair enzyme, O^6^-methylguanine-DNA-methyltransferase (*MGMT*), have been added to the pool of known markers of the glioma progression (Reifenberger et al., 2016). These constant advancements are helpful tools for the diagnosis of the disease but the treatment efficiency and options remain limited. Due to the existence of many different subtypes, we focused on the GBM subtype and summarized the features of the disease in Figure 1.

In the last decade, research has intensified on deciphering the complexity of this devastating disease, yet there is no cure for GBM. The current treatment for glioma is a combination of chemotherapy using Temozolomide and irradiation after resection of the tumor. Despite this radical procedure the average survival increases by 3 months only (Stupp et al., 2005). The detrimental prognosis of glioma is due to the high aggressiveness of the disease where recurrence of the tumor after its resection commonly occurs. Gliomas can use their environment to grow and invade very quickly the healthy brain. Recent findings demonstrate the ability of glioma cells to seize control by the incorporation into the neuronal network of the brain (Venkatesh et al., 2019). Peripheral microglia are attracted and recruited inside the tumor core by the secreted factors released from the tumor cells like CCL2, CSF1, or EGF (Nolte et al., 1997; Zhang et al., 2012; Pyonteck et al., 2013; Sielska et al., 2013). These tumoral environment enrolled microglia will turn into pro-tumoral cells displaying an opposing function to their protective brain homeostatic role. This is evident by the secretion of factors involved in extracellular matrix degradation like MMPs or in angiogenesis like VEGF, EGF, or IL1B (Tsai et al., 1995; Lafuente et al., 1999; Markovic et al., 2009), which are needed for tumor growth and the invasion into the healthy tissue.

Even though the study of glioma-associated microglia is challenging due to the lack of specific microglia markers and the infiltration of peripheral myeloid cells, a few human and mouse studies have defined up-regulated markers linked with microglia/macrophages in the glioma context. Both, CD163 and CD204, identify anti-inflammatory glioma-associated microglia/macrophages (or GAMs), which are present in higher glioma grade and consequently leading to worse survival (Komohara et al., 2008). A recent gene expression meta-analysis on two different glioma mouse models by Haage et al. (2019) distinguished microglia from macrophages in the healthy brain and under glioma conditions. Using publicly available mouse datasets, the authors identified in line with other studies (Hickman et al., 2013; Butovsky et al., 2014; Bennett et al., 2016) *P2ry12, Tmem119, Slc2a5*, and *Fcrls* as a set of genes specific to microglia that will help to differentiate these from macrophages as has been validated in RCAS and GL261 glioma mouse models (Haage et al., 2019). This study confirmed previous data obtained by Bowman et al. (2016) where, using lineage tracing mouse models, they observe that macrophages and microglia would acquire specific transcriptional networks through a tumor-mediated education.

## Microglia in AD

Alzheimer’s disease is the most common neurodegenerative disease with a higher prevalence in women (Figure 1) and clinically defined by the gradual decline in memory and other cognitive functions. Regionally, AD begins in the medial temporal lobes and progresses in a caudal–rostral manner to the frontal lobe (Figure 1). Pathologically, it is characterized by neuroinflammation, extensive neuronal loss, and the progressive accumulation and deposition of insoluble amyloid β (Aβ) plaques in the brain. Plaques have long been considered as a causal effect to AD initiation, yet this hypothesis is under debate (De Strooper and Karran, 2016). The amyloid hypothesis was fueled by the identification of a dominant inherited genomic alteration in three genes resulting in imbalanced processing and subsequent aggregation of Aβ. Mutations in *APP*, *PSEN2*, and *PSEN2* as well as the triplication of the *APP* gene contribute to 5–10% of early onset familial forms of AD. However, familial early onset AD is extremely rare and maybe an atypical form of the disease, representing 1–2% of all cases (Campion et al., 1999).

Aging and genetic components are the two main risk factors for AD with the vast majority of patients displaying AD symptoms from 65 years of age with an average survival time of 8.3 years from diagnosis (Figure 1; Alzheimer’s Association, 2019). Although this late onset AD (LOAD) involves a strong genetic predisposition, no single model can explain the mode of disease transmission. To date, more than 30 gene loci have been implicated in LOAD with the *APOE* gene being the major risk factor (Corder et al., 1993). Several other low-risk loci have been implicated in LOAD including *TREM2*, *CLU*, *PICALM*, *CR1*, *BIN1*, *MS4A* gene cluster, *CD2AP*, *CD33*, *EPHA1*, and *ABCA7* (Harold et al., 2009; Lambert et al., 2009; Hollingworth et al., 2011; Naj et al., 2011; Kunkle et al., 2019). Risk variants of AD that are associated with microglia of the aged brain (Figure 1) are TREM2, *CD33*, *INPP5D*, *HLA-DQA1*, *MS4A4A*, *CASS1*, *SORL1*, *SCIMP*, *SPPL2A*, *APOC1*, and *ATXN7L* (Olah et al., 2018), which makes microglia a central player in recent AD research. Although it is currently not clear how microglia contribute to the disease, various studies point toward inefficient microglial phagocytosis of Aβ plaques and lipid processing (Shi and Holtzman, 2018) as at least one of the main contributors. Microglia are associated with plaques in murine AD models, as well as in AD patients. Current understanding of the role of these plaque-associated microglia indicates that they form a barrier surrounding amyloid deposits, limiting their outward expansion (Yuan et al., 2018).

## Supporting Evidence for the Inverse Relation Between AD and Cancer

Almost three decades ago the first two reports emerged on a negative association regarding the occurrence of cancer of various tissue origin and AD in the same individual (Tirumalasetti et al., 1991; DeSouky, 1992). Both letters state that patients diagnosed with AD did not have cancer or undergone cancer treatment at the time of the investigation. *Vice versa*, cancer patients are less likely to develop AD. Since then a small number of longitudinal studies were conducted all of which reinforce these observations (Yamada et al., 1999; Roe et al., 2005, 2010; Driver et al., 2012; Musicco et al., 2013; Ou et al., 2013). Two independent lines of research revealed that patients with a history of cancer had a reduced risk of developing AD by 33–40%, while dementia patients demonstrated a reduced risk of 56–70% for future cancer (Roe et al., 2010; Driver et al., 2012). Additionally, the quantity of cancer (>1) has shown to lower the chances of AD (Nudelman et al., 2014). Most interestingly, this inverse relationship was greatly affected by the type of dementia. While reportedly vascular dementia demonstrated no significant association to cancer, the likelihood to develop cancer was the lowest in patients with pure or probable AD (Roe et al., 2010; Driver et al., 2012). One of the common risk factors for both diseases is age, which has been taken into consideration by two independent studies. The research conducted in Taiwan reported that especially female patients diagnosed with AD between 60 and 79 years of age had a reduced risk for cancer (Ou et al., 2013). Musicco et al. (2013) revealed a greater decrease on cancer risk in AD patients of both genders from 65 years onward, which is in line with the average begin of diagnosis of AD (Alzheimer’s Association, 2019). In contrast, this study comprised of an Italian cohort suggests that the risk remained low with increasing age to 85+ years (Musicco et al., 2013). The age-related observation in patients with a cancer history exhibiting a smaller likelihood to develop AD was made in the same study. Despite the knowledge that the type of dementia can play a role in the described effect here, nearly all studies applied data of cancer overall and largely not distinguishing between different types of cancer. This is due to the high variability in the prevalence of various cancers. Our interest lies in the relationship between AD and glioma and more specifically the role of microglia in this association. The current challenge is the collection of sufficient and significant information on the relationship between glioma and AD considering the low incidence rate of primary brain tumors such as GBM (∼2–3 in 100,000).

## Microglia Associated Genes and Pathways as Possible Players in Disease Fate

The underlying mechanism and/or genetic predisposition that support the pathology of one but not the other disease has been speculated (Behrens et al., 2009; Liu T. et al., 2013; Snyder et al., 2017), yet the question what initiates the fate toward either condition remains. What came first? Glioma or AD and what specific role could microglia play in this correlation? In the absence of disease, age-related changes of the microglia transcriptome are apparent as early as middle-age and regionally distinct. Particularly, changes in sensing and engaging with their local environment correlate with the regional susceptibility to age-related neurodegenerative diseases (Grabert et al., 2016). While AD is diagnosed for the majority of cases from 65 years of age (Alzheimer’s Association, 2019), the development of the disease and early markers (e.g., CSF, Aβ) has its beginnings decades before (Jack and Holtzman, 2013; Selkoe and Hardy, 2016). Thus, as AD progresses over years prior to the clinical diagnosis the local environment may be pushed in one direction and thereby restrain the development of glioma. In contrast, the prevalence of glioma increases drastically at the age of 45 years (Ferris et al., 2017). Cells (e.g., microglia) of cancer survivors may have acquired a distinct phenotype throughout the disease and treatment which prevents the development of AD. Here we describe a small number of selected genes and pathway related to microglia in either disease, which we think could be of interest for future investigations and application.

TREM2 is a cell surface receptor highly expressed in microglia. It recognizes multiple components including apoptotic cells, apolipoproteins (e.g., APOE and CLU), Aβ oligomers (Zhao et al., 2018), and endogenous ligands such as galectin-3 (Boza-Serrano et al., 2019). Activation of TREM2 leads to the sequestration of DAP12 leading to microglial proliferation, survival, and phagocytosis (reviewed by Shi and Holtzman, 2018). Notably, APOE, CLU, and DAP12 are identified as AD risk genes (Figure 1). The role of TREM2 in AD has been the subject of intense research. TREM2 expression is upregulated in microglia with the progression of the disease, when microglia feature an enhanced phagocytotic profile (Keren-Shaul et al., 2017). TREM2-deficient microglia show reduced Aβ uptake *in vitro* (Yeh et al., 2016), *in vivo* (Wang Y. et al., 2016; Yuan et al., 2018), as well as reduced aggregation and proliferation around plaques (Jay et al., 2015; Wang et al., 2015). On the other hand, a recent study reported that TREM2 is significantly upregulated in glioma tissue and is associated with glioma progression (Wang X.-Q. et al., 2016). Further studies to elucidate the role of TREM2 in AD and glioma and its relation to microglia are needed.

APOE is a lipid carrier regulating lipid homeostasis and also acts as a ligand for TREM2, regulating the TREM2-mediated microglia phagocytosis (Yeh et al., 2016). There are three common human APOE alleles, ε2, ε3, and ε4, which differ by two amino acids. APOE allele ε4 is the major known genetic risk factor for AD; it is estimated that 40% of people diagnosed with AD have this allele (Liu C.-C. et al., 2013). In contrast, APOE ε3 appears not to have a correlation with AD, while APOE ε2 has been shown to infer a protective role (Yeh et al., 2016). If APOE should have a role in glioma, it has not yet been revealed.

Neuropillin-1 (or NRP1), a transmembrane receptor expressed by various cells in the body, plays a role in brain tumor progression through its expression in microglia. Indeed, Miyauchi et al. (2016, 2018) demonstrated that the deletion of NRP1 in microglia leads to the reduction of glioma progression *in vivo* and its expression in microglial cells is correlated with poor prognosis in high grade gliomas (Caponegro et al., 2018). The function of NRP1 in AD is poorly studied with only one study hinting toward NRP1 as potential marker of inflammatory microglia in AD based on the proteomics analysis of microglial plasma membranes after treatment with a synthetic Aβ peptide (Correani et al., 2017).

Our group uncovered the non-apoptotic role of Caspase-3 (CASP3) in the phenotype polarization of microglia. The stimulation of microglia cells by inflammogens will lead to the activation of CASP3 without cell death induction *in vitro* or *in vivo*. In turn, active CASP3 promotes, through a PKCδ-dependent pathway, the pro-inflammatory activation of microglia. This microglial *CASP3* activation has been observed in the frontal cortex of AD patients which confirms a role for CASP3 in AD (Burguillos et al., 2011). In contrast, the activation of microglia into a pro-tumoral phenotype under glioma conditions is associated with a decrease of the basal level of CASP3 in microglia by the glioma cells (Shen et al., 2016). These opposite roles of CASP3 in the different microglial phenotypes acquisition between AD and glioma could be an explanation of the inverse correlation of the two pathologies and the role microglia could play in the fate of the brain (for review see Shen et al., 2017, 2018).

A few studies have highlighted the phosphoinositide 3 kinase – Akt – mammalian target of rapamycin (PI3k-Akt-mTor, PAM) pathway (Liu T. et al., 2013; Majd et al., 2019) as a possible common mechanism that may be the link in the inverse relation between AD and cancer in general. Thus, proteins upstream or downstream of this pathway could exert influence. Interestingly, some of the example genes described here can partly be linked with PAM underlining a potentially critical role in the decision process for either disease. In case of TREM2, upon ligand binding (e.g., APOE) and interacting with DAP12 as adapter protein the PI3K cascade is activated leading to cellular proliferation, survival, and phagocytosis (Konishi and Kiyama, 2018). With regards to AD, this mechanism is thought to support highly energetic microglia in order to cluster around Aβ plaques (Zhou et al., 2018). Yet it could be identified that the microglial cells of AD patients with a confirmed deficiency in TREM2 trigger the enhancement of mTOR-regulated autophagy for survival (Ulland et al., 2018).

In conclusion, these few examples demonstrate the current lack of information in the interplay of microglia-associated genes and pathways toward either pathology. Less than a handful of genes are well described to be active in AD (e.g., CASP3) but decrease in glioma; or upregulated in glioma and being a risk variant in AD leading to gene deficiency (e.g., TREM2). Yet, some genes could be both up- or downregulated in either disease due to the effect of aging. CLEC7A (C-type lectin domain containing 7a, also known as DECTIN-1) is one example. This anti-microbial pattern recognition receptor (Goodridge et al., 2011) is increasingly expressed by microglia with age (Holtman et al., 2015; Raj et al., 2017). Moreover, the expression of *Clec7a* in mouse models of neurodegeneration (e.g., multiple sclerosis, amyotrophic lateral sclerosis, AD) (Olah et al., 2012; Keren-Shaul et al., 2017; Krasemann et al., 2018) and glioma (Szulzewsky et al., 2015) was upregulated and has recently included as a marker for disease-associated microglia (DAM) (Keren-Shaul et al., 2017). Thus, it is necessary to extend the scope of future research for which we provide a number of suggestions in the subsequent section.

## Proposed Guidelines for Future Research and Diagnostics

**FIGURE 2:**
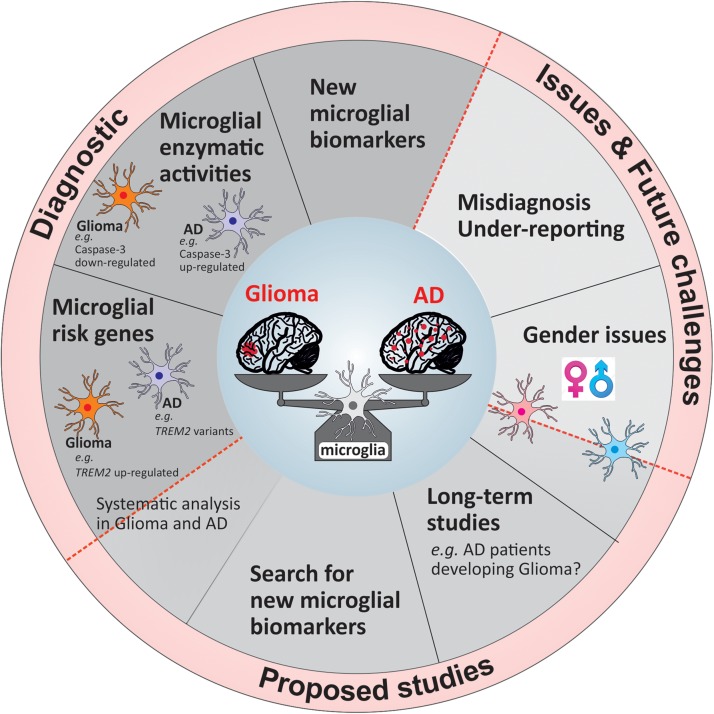
Overview of the current challenges and proposed guidelines for future studies and diagnostic tools.

To uncover and understand the microglial and mechanistic contribution to the inverse relation of AD and glioma, future research faces a number of challenges, which once overcome can lead to new tools in diagnostics and ultimately alternative treatment opportunities. In Figure 2, we summarized our suggestions for future studies in a form of potential guidelines that could be followed either in research investigations or in a clinical setting.

The majority of longitudinal studies examined the relation between cancer and AD on a broad scale largely unable to concentrate on the association of AD with a distinct type of cancer. Certainly, the analysis into the relation of glioma with AD will take a considerable number of years due to the low frequency occurrence of glioma. A large-scale study stretching over a significant period of time would further allow the distinction between men and women (Figure 2) as has been reported that women are more likely to develop AD than cancer, while this is the opposite for men. Moreover, the evidence regarding the differences of microglia based on gender is increasing (Guneykaya et al., 2018; Villa et al., 2018) underlining the importance of distinction. One issue of previous and possibly future studies will be the misdiagnosis of glioma for AD and *vice versa* (Figure 2) as initial pathology with the performance of minimal test to establish correct diagnosis as likely reason. Furthermore, once a patient is diagnosed with either disease no additional examination is being performed to exclude the possibility of the other. Thus, either disease may remain undetected and therefore is not being reported (Figure 2). Future work, although laborious and costly, would profoundly benefit from extended examination to underline present findings. Simple tools such as diagnostic marker would facilitate this procedure.

The use of microglial markers that are expressed in one pathology and not the other will be of great benefit for the diagnosis at early stages of the diseases. As mentioned previously, a marker like Caspase-3 or disease-associated risk alleles, which are expressed differentially in microglia based on the disease context would be a highly valuable tool to add in the first steps of the patients’ clinical investigation. The challenge to identify genes or proteins that could be applied in the clinical setting is due to the high percentage of studies focusing on few of many microglia-associated risk genes in AD in great detail (e.g., TREM2, APOE). Simultaneously, identified AD risk genes are not or weakly studied in a different disease setting such as glioma. Considering the remarkable inverse relationship of these pathologies it could be worth to investigate distinct risk genes greatly associated with one disease regarding their nature in the other. There is an obvious need to identify other potential microglial targets useful for diagnostics which could be done by developing a larger scale study including AD and glioma patients.

In summary, there is very little knowledge and evidence how microglia may contribute toward the fate of these two opposing pathologies. More than a decade ago, Schwartz et al. (2006) reviewed the idea whether stimulated microglia may be set for a distinct phenotype or if there is the possibility to reverse an induced functional phenotype. It was concluded that based on the type of the primary and secondary trigger, microglia are capable either to reverse or exaggerate their commitment. It is then possible to consider that once activated in a specific disease context, microglia would maintain this acquired disease phenotype (e.g., AD) and could be unable to go back to another state, excluding the apparition of another disease such as glioma.

## Author Contributions

MC, VS, BJ, and KG planned and wrote the manuscript.

## Conflict of Interest

The authors declare that the research was conducted in the absence of any commercial or financial relationships that could be construed as a potential conflict of interest.
